# Antipsychotic use and associating factors among persons with substance-induced psychosis and first-episode psychotic disorders. A nationwide register-linkage study

**DOI:** 10.1192/j.eurpsy.2024.716

**Published:** 2024-08-27

**Authors:** J. Jeyapalan, S. Niemela, H. Taipale

**Affiliations:** ^1^Department of Psychiatry, University of Turku, Turku; ^2^Department of Psychiatry, Vaasa Central Hospital, Vaasa; ^3^Department of Psychiatry, University Hospital Turku, Turku, Finland; ^4^Clinical Neuroscience, Karolinska Institute, Stockholm, Sweden; ^5^Niuvanni Hospital, Kuopia; ^6^Neuroscience Centre, University of Helsinki, Helsinki, Finland

## Abstract

**Introduction:**

Far less is known about the preceding factors of antipsychotic use among persons with substance-induced psychosis (SIP) and first-episode psychosis (FEP). There is no prevention research on how persons with SIP differ from persons with other psychosis episodes like FEP. Antipsychotic medication is the general essential and necessary element in the treatment of SIP and FEP1. Antipsychotics are used as first-line therapy, commencing with a low dose and titrating upwards2. There are no exciting treatment guidelines for treating Substance-induced psychosis in the long term. (A review of some studies published by the Oxford Journals Schizophrenic Bulletin indicated that drug-induced psychosis lasted longer than a month in individuals between 1 and 15% of the time.3)

The aim of the study was to investigate antipsychotic use and associated factors in persons with SIP and compare it with persons with other FEP

**Objectives:**

1 To study the antipsychotic use among persons with SIP compared with FEP from 3 years before until three years after their first diagnosis first incident of psychosis)

2.To study associating background factors with antipsychotic use among patients with SIP

**Methods:**

Incident Swedish SIP cases (n=7320)during 2006-2016 were identified from health care registers and matched 1: with persons with FEP (n=7320) by age, gender, and calendar year of diagnosis. Prevalence of antipsychotic use was assessed as point prevalence every six months, from 3years before until 3years after the first diagnosis. Factors associating with antipsychotic use among SIP were analyzed with multivariable logistic regression, including information on sociodemographic and work-related background, including disability pension and sickness absence, SIP types, and psychiatric diagnoses.

**Results:**

Among SIP and FEP, the prevalence of antipsychotic use was low before the first diagnosis (3-7% in SIP, 8-16% in FEP), peaked 6 months after the first diagnosis (23% in SIP, 54% in FEP) and stabilized after that. After 3 years of first diagnosis, 19% of persons with SIP and 45% of persons with FEP used antipsychotics. Antipsychotic use one year after diagnosis among SIP was associated with previous substance use disorder, depression, anxiety, and personality disorder diagnoses, being on disability pension or on long-term sickness absence (>90 days), and cannabis- or multi-substance-induced psychosis.

**Image:**

**Figure:**
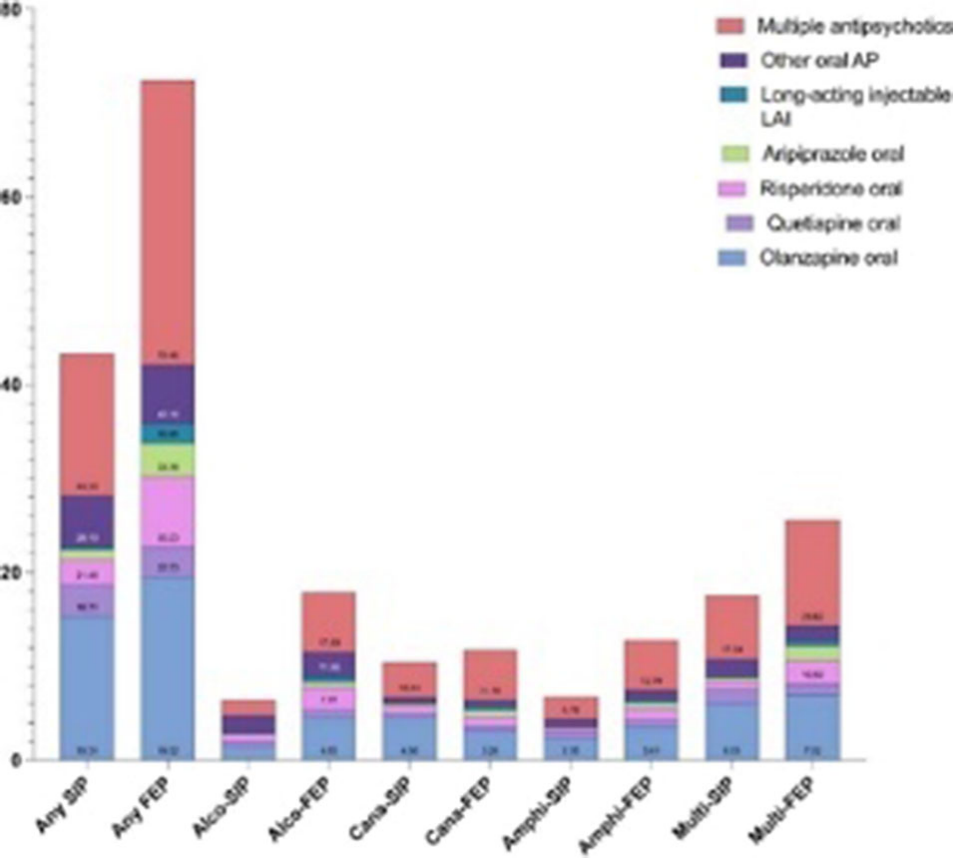

**Image 2:**

**Figure:**
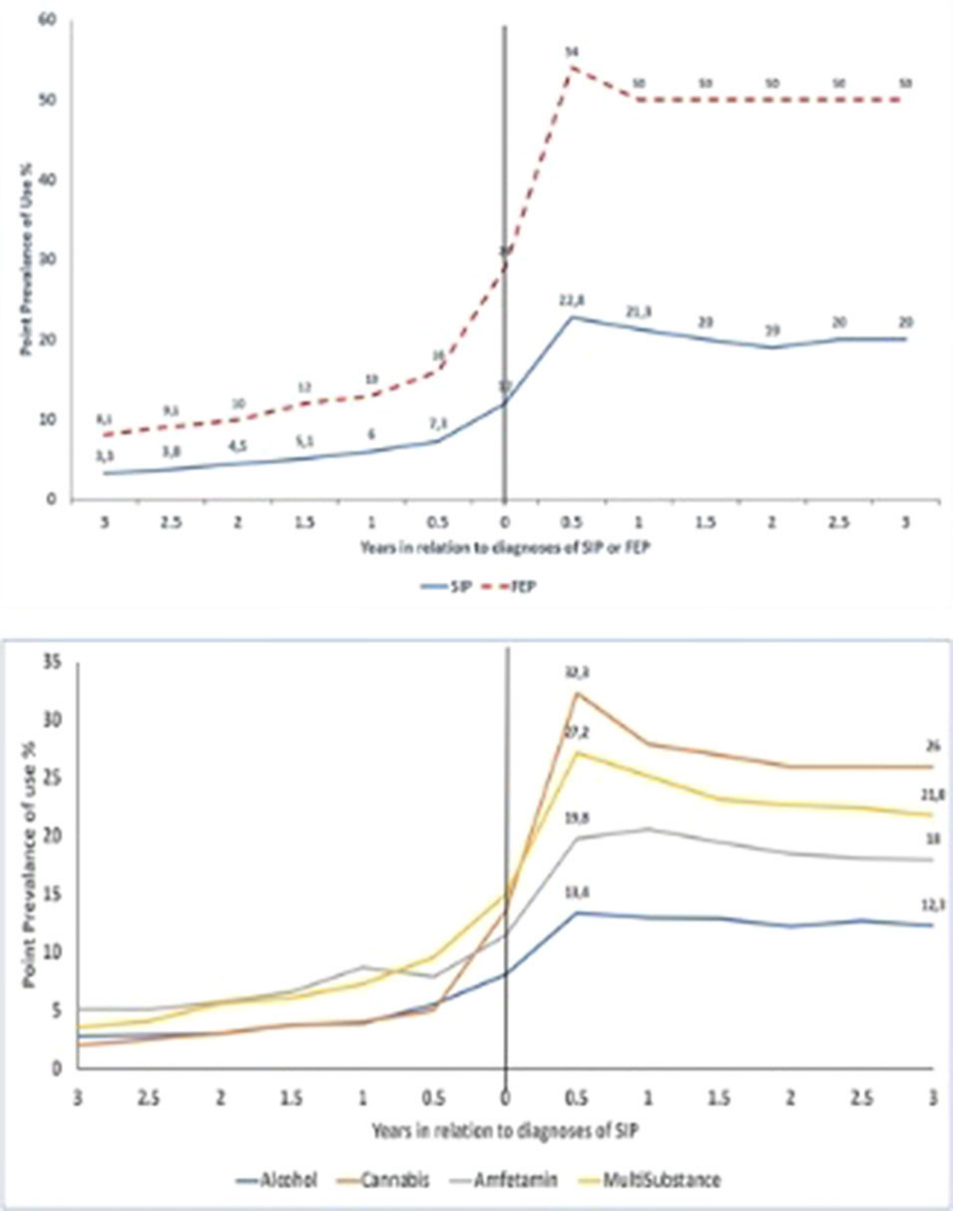

**Conclusions:**

As expected, patients with FEP were using more frequently antipsychotics compared to SIP except for long-acting antipsychotics.

Although SIP is considered short-lived, antipsychotic use after an incident SIP episode is relatively common, especially among those with cannabis SIP with the highest prevalence of antipsychotic use.

Previous substance use disorder and cannabis SIP were highly associated with patients who use antipsychotics frequently.

**Disclosure of Interest:**

None Declared

